# Imaging patients with breast and prostate cancers using combined 18F NaF/18F FDG and TOF simultaneous PET/ MRI

**DOI:** 10.1186/2197-7364-2-S1-A65

**Published:** 2015-05-18

**Authors:** Andrei Iagaru, Ryogo Minamimoto, Mehran Jamali, Amir Barkodhodari, Sanjiv Sam Gambhir, Shreyas Vasanawala

**Affiliations:** Stanford University, Department of Radiology, Division of Nuclear Medicine and Molecular Imaging, USA

Here we prospectively compared the combined 18F NaF/18F FDG PET/ MRI against 99mTc-MDP in patients with breast and prostate cancers. Twelve patients referred for 99mTc-MDP bone scans were prospectively enrolled from Oct 14 - Jan 15. The cohort included 6 men with prostate cancer and 6 women with breast cancer, 41 – 85 year-old (average 63 ± 15). 18F NaF (0.7-2.2 mCi, mean: 1.33 mCi) and 18F FDG (3.9-5.2 mCi, mean: 4.6 mCi) were subsequently injected from separate syringes. The PET/MRI was done 6-12 days (average 9.3 ± 3.2) after bone scan. The whole body MRI protocol consisted of T2-weighted, DWI, and contrast-enhanced T1-weighted imaging. Lesions detected with each test were tabulated and the results were compared. All patients tolerated the PET/MRI exam, and PET image quality was diagnostic despite the marked reduction in the administered dosage of radiopharmaceuticals (80% less for 18F NaF and 67% less for 18F FDG). Five patients had no bone metastases identified on either scans. Bone scintigraphy and PET/MRI showed osseous metastases in 7 patients, but more numerous bone findings were noted on PET/MRI than on bone scintigraphy in 3 patients. Lesions outside the skeleton were identified by PET/MRI in 2 patients. The combined 18F NaF/18F FDG PET/MRI is superior to 99mTc-MDP scintigraphy for evaluation of skeletal disease extent. Further, it detected extra- skeletal disease that may change the management of these patients, while allowing a significant reduction in radiation exposure from lower dosages of PET radiopharmaceuticals administered. A combination of 18F NaF/18F FDG PET/MRI may provide the most accurate staging of patients with breast and prostate cancers prior to the start of treatment.

